# Hidden in Plain Sight: Deciphering Chest Pain, Hypertension, and Elevated Creatinine to Unveil Aortic Dissection

**DOI:** 10.7759/cureus.65892

**Published:** 2024-07-31

**Authors:** Karumanchi Sudeepa, Mythili K, Nemani Sai Manasa, J. S Kumar, Nirmala C

**Affiliations:** 1 Internal Medicine, Sri Ramaswamy Memorial Institute of Science and Technology (SRMIST), Chennai, IND; 2 General Medicine, Sri Ramaswamy Memorial Institute of Science and Technology (SRMIST), Chengalpattu, IND; 3 Internal Medicine, Sri Ramaswamy Memorial Institute of Science and Technology (SRMIST), Chengalpattu, IND

**Keywords:** multidisciplinary approach, hypertension, medical management, stanford type b, aortic dissection

## Abstract

Chest pain is a common yet complex presentation in the emergency department, often requiring the exclusion of life-threatening conditions such as aortic dissection. Stanford type B aortic dissection, which affects the descending aorta, poses significant diagnostic and therapeutic challenges but can often be managed medically without immediate surgery. This case underscores the necessity of having a vigilant mindset, performing a detailed clinical examination, and including aortic dissection in the differential diagnosis, especially when typical symptoms are observed. The challenging part of this case was the investigation, as computed tomography angiography couldn't be performed, necessitating the use of magnetic resonance imaging for diagnosis. It highlights the importance of individualized patient care, vigilant monitoring, and comprehensive management strategies in the treatment of aortic dissection.

## Introduction

In the emergency department (ED), evaluating chest pain is complex due to the variety of ways it can manifest, influenced by factors like the patient's age, gender, and other health conditions. While often stemming from non-cardiac sources such as acid reflux or musculoskeletal issues, it's essential to eliminate serious possibilities such as myocardial infarction, pulmonary embolism, visceral perforation, aortic dissection, and tension pneumothorax. Acute aortic dissection (AAD) is a rare, life-threatening condition caused by a tear in the aortic wall intimal layer, allowing blood to separate the intima and media. This dissection can progress either proximally or retrograde, resulting in high mortality, with many patients dying before arriving at the emergency department. AAD typically presents with abrupt, intense tearing type of chest pain, but symptoms can be subtle, leading to frequent misdiagnoses [1,2]. Untreated, the mortality rate can reach 50% within the first 48 hours [3]. AAD is classified using two systems: the Stanford system, more widely used, classifies aortic dissections as type A, which involves the ascending aorta, and type B, which involves only the descending aorta. The older De-Bakey system classifies dissections as type I, which begins in the ascending aorta and extends to all aortic segments; type II, which is confined to the ascending aorta; and type III, which affects only the descending aorta. Ascending aortic dissections are nearly twice as common as descending ones. High-risk factors for non-traumatic AAD include hypertension (present in 70% of distal type B cases)-Sudden, severe increase in blood pressure (e.g., heavy lifting, stimulant use), genetic conditions (e.g., Ehlers-Danlos), existing aortic aneurysm, arteriosclerosis, family history, and prior aortic surgery or instrumentation [4-6].

## Case presentation

An Indian male patient, aged 45, without any known underlying conditions, presented to the Sri Ramaswamy Memorial (SRM) emergency department reporting complaints of central sharp chest pain radiating abruptly in onset to the back between the shoulder blades lasting for 10-15 minutes and shortness of breath for 15-20 mins. Upon examination, his blood pressure in the left upper limb was 220/140 mmHg in the supine posture, and in the right upper limb was 210/130 mmHg taken in the supine position, pulse rate was recorded at 80/min, and no pulse deficits were noted, and his respiratory rate was 20 breaths per minute. On auscultation, the second heart sound in the aortic area was loud, and while the first heart sound was normal, no murmurs were detected. Laboratory tests were subsequently ordered, and the results are summarized in Table 1.

**Table 1:** Key laboratory parameters

Parameter	Value	Reference range
Hemoglobin (Hb)	12 gm/dL	13.5-17.5 gm/dL (male), 12.0-15.5 gm/dL (female)
Hematocrit (Hct)	36%	41%-50% (male), 36%-44% (female)
White blood cell count (WBC)	4500/microliter	4000-11,000/microliter
Platelets	230,000/UL	150,000-450,000/UL
C-reactive protein (CRP)	0.8 mg/dL	<1.0 mg/dL
Random blood sugars	120 mg/dL	70-140 mg/dL
Serum creatinine	2.5 mg/dL	0.6-1.2 mg/dL (male), 0.5-1.1 mg/dL (female)
Blood urea	20 mg/dL	7-20 mg/dL
Troponin I (initial)	0.001	<0.01 ng/mL
Troponin I (after three hours)	0.002	<0.01 ng/mL
Creatinine kinase - MB (CK-MB)	17	0-24 IU/L
Total creatinine kinase (CK)	117	Male: Up to 171 IU/L, female: Up to 145 IU/L
Urine routine	Protein 1+, No RBCs, No WBCs, granular cast +	
24-hour urine protein	110.4 mg/dL	Less than 150 mg/day
Urine spot polymerase chain reaction (PCR)	1.36	<0.2

**Figure 1:**
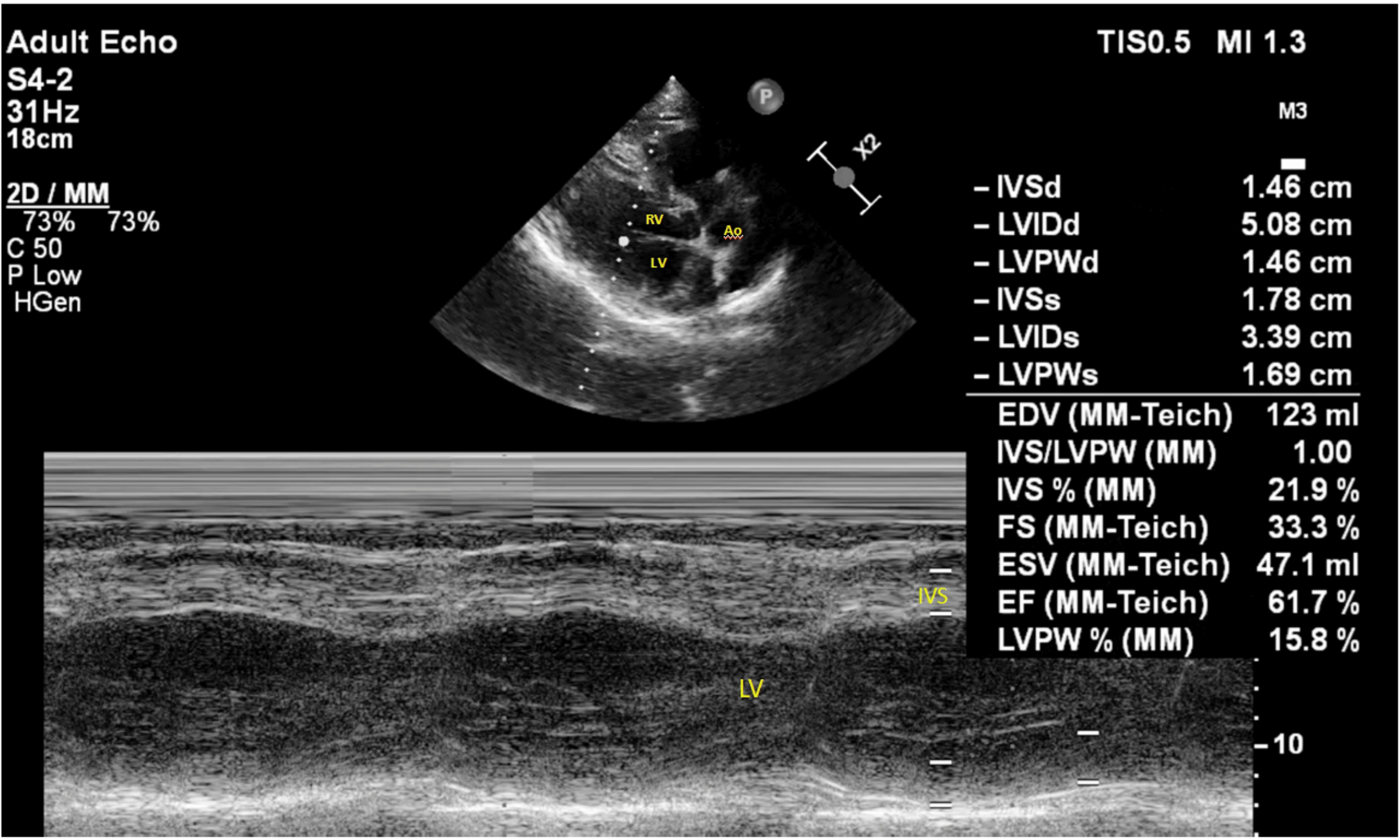
2D echocardiogram demonstrating left ventricular hypertrophy LV - left ventricle; RV - right ventricle; Ao - aorta; IVS - interventricular septum

**Figure 2:**
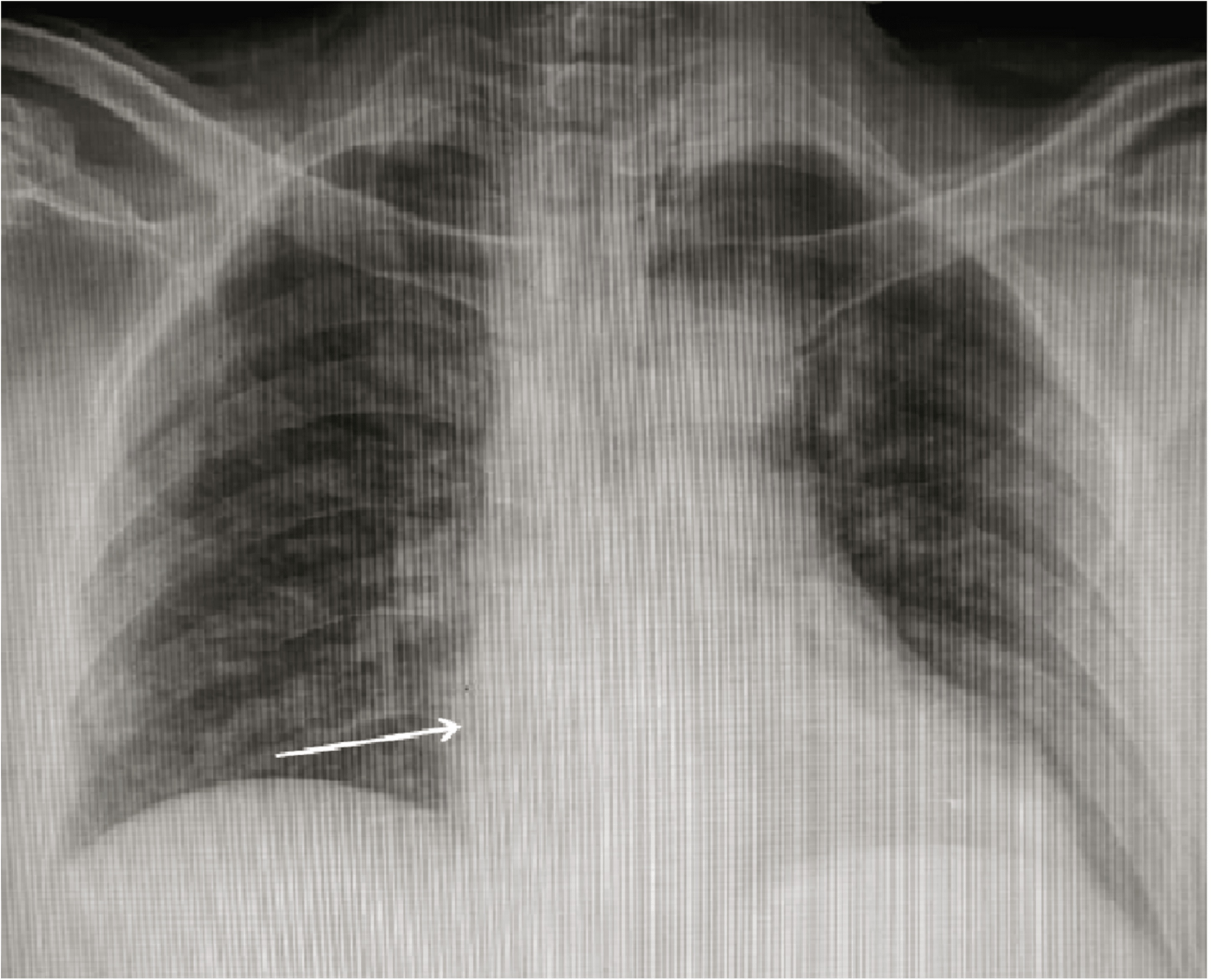
Chest X-ray anterio-posterior view suggestive of apparent cardiomegaly

The electrocardiogram (ECG) showed left ventricular hypertrophy (LVH), heart rate (HR) of 78/min with normal sinus rhythm, and a 2D echocardiogram was performed, showing left ventricular hypertrophy (LVH), no regional wall motion abnormalities, an ejection fraction of 63% (Figure 1). A chest X-ray antero-posterior view was performed, which was suggestive of apparent cardiomegaly (Figure 2). 

The patient spent an initial two hours in the emergency department for stabilization, where intravenous (IV) labetalol was administered, followed by an IV infusion of nitroglycerin, and was then transferred to the ICU for close monitoring until blood pressure was controlled.

In the ICU, the IV infusion of nitroglycerin was continued for 20 hours and slowly tapered, overlapped with tablet metoprolol, and then stopped. Blood pressure was frequently monitored, and medication dosages were adjusted accordingly. For pain management, IV morphine 2 mg was given. Due to the patient's increasing creatinine levels and persistent elevated blood pressures, a renal artery Doppler ultrasound was performed, which yielded normal results.

**Figure 3:**
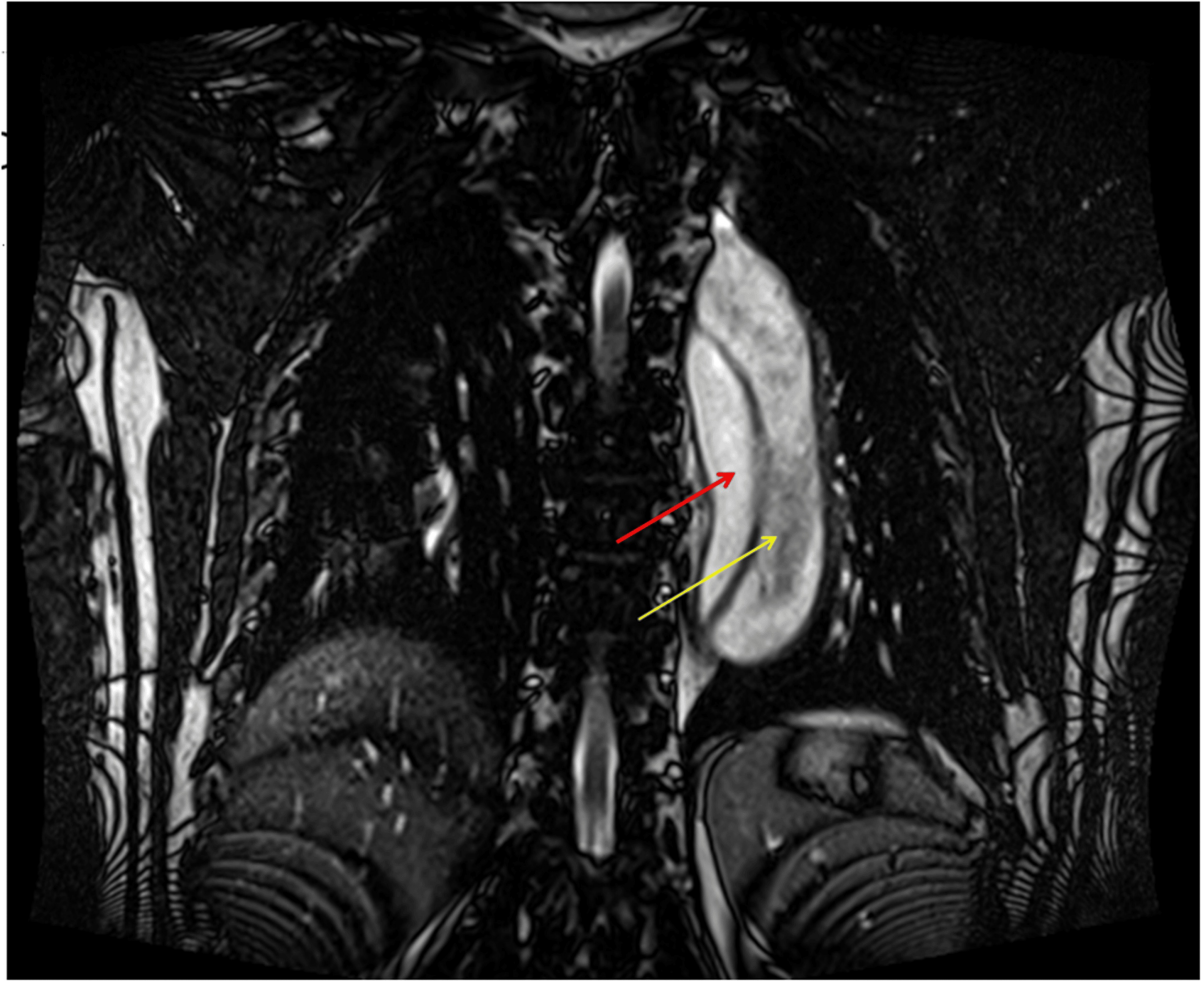
T2-weighted MRI coronal view that demonstrates a dissection of thoracic aorta (approximately 4 cm in diameter) with a smaller true lumen (red arrow) measuring 1.5cm positioned medially, while the larger false lumen (yellow arrow) measuring 2.5 cm positioned laterally

**Figure 4:**
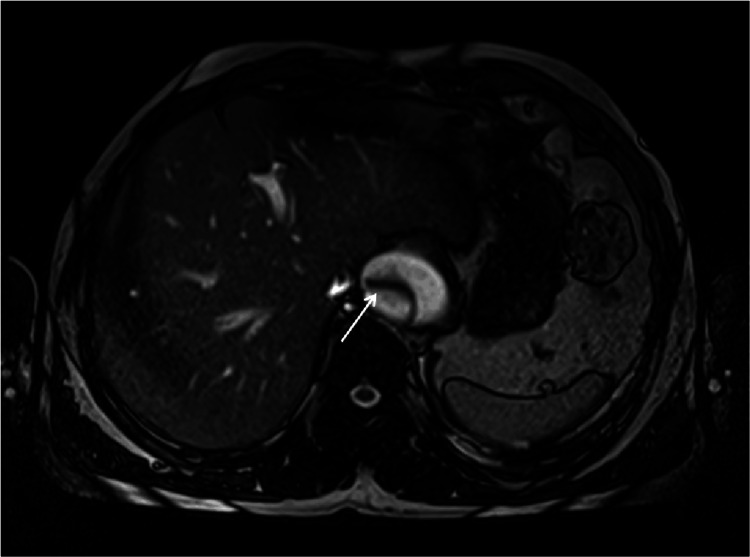
T2-weighted MRI - axial section at the level of liver highlighting an aortic dissection

Despite ongoing treatment, he continued to experience persistent inter-scapular pain. Based on the clinical information, laboratory tests, chest X-ray, and physical examination findings, an aortic dissection was suspected. Due to abnormal renal function tests, computer tomography angiography was not feasible. Instead, magnetic resonance imaging (MRI) of the chest and abdomen was conducted, which confirmed a thoracic aortic dissection classified as grade III according to the De-Bakey system and type B per the Stanford classification (Figures 3, 4).

A multidisciplinary team, including cardiologists, vascular surgeons, and radiologists, was consulted for optimal management. Considering the extent of the type B aortic dissection, the cardiothoracic team predicted the surgical risk to be high. Additionally, the patient preferred conservative treatment over surgery. The patient was placed on stringent blood pressure control with a target of 120/80 mmHg and a heart rate target of 60/minute using tablet metoprolol 50 mg twice daily and tablet glyceryl trinitrate 2.6mg thrice daily. Antiplatelet therapy with aspirin and statins was initiated. For pain management, IV morphine 2 mg was administered as needed (SOS) during the hospital stay, successfully bringing the patient's blood pressure to 120/80 mmHg and heart rate to 62/minute.

After stabilization of blood pressure, the patient was transferred to the ward for further monitoring and management.

At discharge, an antihypertensive regimen comprising of metoprolol and glyceryl trinitrate was advised to continue. Regular follow-up imaging every three to six months, then annually if stable, was recommended to monitor the dissection and aortic diameter for complications such as aneurysm formation or progression of the dissection.

During follow-up visits, both home and office blood pressure readings remained well-controlled, with baseline measurements consistently around 130/80 mmHg. Follow-up lab tests indicated a decrease in the patient’s plasma creatinine level, now at 2.3 mg/dL. A contrast CT angiography was planned once the creatinine levels approached near-normal values.

The patient was also advised on lifestyle modifications, including a heart-healthy diet, regular physical activity, and avoiding heavy lifting or strenuous activities that could elevate blood pressure.

## Discussion

In this case, the patient's persistently elevated blood pressures and chest pain radiating to the back, coupled with normal cardiac markers, raised suspicion for aortic dissection. Despite the complexities posed by the patient's deranged renal function, which made CT angiography unsuitable, an MRI was successfully employed to confirm the diagnosis. This emphasizes the importance of keeping a high level of suspicion and considering aortic dissection in the differential diagnosis when classical symptoms are evident.

Physical examination can offer crucial insights for diagnosing aortic dissection. It is recommended to measure blood pressure in both arms and legs, noting that elevated blood pressures occur in 70% of distal dissections and 35% of proximal ones. Additionally, checking pulses in all four limbs is essential, as less than 20% of patients may present with a pulse deficit or difference, often caused by an flap from intimal layer or hematoma leading to compression Furthermore, about half of the patients with proximal aortic dissections exhibit a diastolic murmur suggestive of aortic regurgitation, which can sometimes be faint and potentially overlooked by physicians [7].

The patient's elevated creatinine levels, despite normal renal artery doppler findings, suggest other mechanisms, such as smaller emboli or debris from the dissected aorta, might have caused micro-infarctions in the renal parenchyma, hypo-perfusion caused by an obstructive dissection flap, as well as relative hypotension from strict blood pressure control, impairing kidney function. Elevated serum creatinine, along with granular cast and tubular range proteinuria in this patient, suggests acute tubular necrosis (ATN) secondary to hypo-perfusion. Renal mal-perfusion remains an elusive diagnosis, necessitating vigilant monitoring [8][9].AKI is believed to be associated with adverse outcomes among acute type B aortic dissection patients and has been reported to occur at a rate between 13% and 36% [10]. Table 2 summarizes case reports of aortic dissection with renal involvement and elevated creatinine levels.

**Table 2:** Summary of case reports on aortic dissection with renal involvement and elevated creatinine levels

Patient age	Gender	Presentation	Type of aortic dissection	Elevated creatinine (yes/no)	Cause for elevated creatinine	Authors and year
65	Female	Severe chest pain radiating to the back, hematuria, crushing chest pain improved when laying on left side, nausea, vomiting, diaphoresis	Stanford type A	Yes	Renal ischemia due to decreased blood flow	Mohamed et al., 2022 [11]
50	Male	Severe central chest pain radiating to left shoulder and neck, sharp then dull pain, no relief with paracetamol and oral morphine	Stanford type A	Yes	Renal hypoperfusion from right renal artery in false lumen	Anene, 2022 [12]
79	Female	Bradycardia, acute chest pain, generalized weakness, pulsatile chest pain after walking, no radiating pain	Type A dissection	Yes	Not clearly mentioned	Skerritt et al., 2019 [13]
63	Female	Chronic cough, chest pain radiating to left scapula, lightheadedness, hoarseness, swallowing difficulty, weight loss	Stanford A	Yes	Not clearly mentioned	Muhammad et al., 2021 [14]
17	Male	Sudden onset of left flank and abdominal pain, coma, hematemesis, hematuria, passage of bloody stool	Stanford type B	Yes	Severe acute renal failure	Ngan et al., 2006 [15]

Considering the patient's high surgical risk and preference for conservative management, our approach aligns with evidence showing that conservative treatment for acute uncomplicated type B aortic dissection has a low 30-day mortality rate of 2.4%. This approach has been shown to be effective, with nearly 90% of patients surviving initial hospitalization and 50-80% surviving five years with medical management [16].

## Conclusions

This case emphasizes the importance of considering aortic dissection in patients who present with chest pain, elevated blood pressure, and renal impairment. In our case, due to the risk of contrast-induced nephropathy (CIN), we opted for magnetic resonance imaging as the diagnostic tool. MRI can be an effective alternative for diagnosing aortic dissection in situations where reducing nephrotoxic risks is crucial. Effective management involved vigilant monitoring and aggressive control of hypertension, illustrating the necessity of a meticulous, multidisciplinary approach in handling complex cases of aortic dissection.
